# In-hospital production of 3D-printed casts for non-displaced wrist and hand fractures

**DOI:** 10.1051/sicotj/2022021

**Published:** 2022-05-24

**Authors:** Shai Factor, Franck Atlan, Tamir Pritsch, Netta Rumack, Eran Golden, Solomon Dadia

**Affiliations:** 1 Department of Orthopedic Surgery, Tel Aviv Medical Center 6 Weizmann St. Tel Aviv 6423906 Israel affiliated with the Sackler Faculty of Medicine, Tel Aviv University Tel Aviv Israel; 2 Surgical Innovation and 3D Printing Unit, Tel-Aviv Medical Center 6 Weizmann St. Tel Aviv 6423906 Israel

**Keywords:** 3-D printing, Digital Light Processing, Wrist fracture, Hand fractures, Patient-reported Outcomes

## Abstract

*Objectives*: To examine the clinical feasibility and results of a multidisciplinary workflow, employing rapid three-dimensional (3D) scanning and modeling software along with a high-speed printer, for in-hospital production of patient-specific 3D-printed casts, for the treatment of non-displaced wrist and hand fractures. *Methods*: Consenting adult patients admitted to the emergency department (ED) due to wrist or hand fractures between January and February 2021 were prospectively enrolled. The study participants underwent conversion of the standard plaster of Paris cast to a 3D-printed cast one week after the ED visit, and follow-up examinations were performed around two, six, and twelve weeks later. The primary objective was to examine the clinical feasibility in terms of complexity and length of the overall procedure. Secondary outcomes were patient-reported impressions and radiological results. *Results*: Twenty patients (16 males, mean age 37 ± 13.1 years) were included. The entire printing workflow took a mean of 161 ± 8 min. All patients demonstrated clinical improvement and fracture union at final follow-up, with no pressure sores or loss of reduction. Patient-reported comfort and satisfaction rates were excellent. The mean Visual Analog Scale was 0.9 ± 1.1 and 0.6 ± 1, and the mean Disabilities of the Arm, Shoulder, and Hand score was 18.7 ± 9.5 and 7.6 ± 7.6 at 2 and 6 weeks after application of the 3D-printed cast, respectively. *Conclusion*: The in-hospital workflow was feasible and efficient, with excellent clinical and radiographic results and high patient satisfaction and comfort rates. Our medical center now routinely provides this cast option for non-displaced wrist and hand fractures.

*Level of evidence*: IV, Therapeutic Study

## Introduction

Three-dimensional (3D) printing technology has increasing relevance and is rapidly advancing in medical treatment [1–5]. There are numerous applications of 3D printing in hand surgery, including patient education, surgical training, preoperative planning, patient-specific surgical guides, and printing of custom-made splints and prostheses [6–9]. 3D-printed orthopedic casts are personalized and fit perfectly to the patients’ anatomy and pathology, improving patient comfort and satisfaction by being light, breathable and washable [10–12]. There is a paucity of data regarding the clinical use of 3D-printed casts, probably due to the complexity and relatively long duration of the procedure. Hoogervorst et al. [13] showed noninferiority of 3D-printed casts compared with the traditional fiberglass casts in immobilizing a subacute distal radius fracture in a cadaver model. Chen et al. [14] performed a clinical trial on the application of a 3D-printed cast for the treatment of forearm fractures and concluded that it was associated with both increased patient comfort and satisfaction, although their study included only ten patients with an age range from 5 to 78 years. Keller et al. [15] published a multidisciplinary workflow for in-hospital mass production of patient-specific 3D-printed devices for hand and wrist rehabilitation. Those authors used intelligible and rapid 3D scanning and modeling software along with an in-hospital high-speed printer. There are no studies examining the clinical use of 3D casts for the treatment of fractures, using a comprehensive work process in one medical center.

The primary objective of this study was to examine the clinical feasibility of in-hospital production of patient-specific 3D-printed hand casts in terms of complexity and length of the overall procedure. The secondary outcomes were patient-reported outcomes and the radiological results.

## Materials and methods

### Study design

Following institutional review board approval, this prospective study was conducted on patients admitted to the emergency department (ED) due to wrist or hand fractures between January and February 2021. Inclusion criteria were adults with non-displaced wrist or hand fractures suitable for conservative treatment with cast immobilization. Excluded were patients who were unable to provide informed consent, those with pathological or open fractures, hypersensitivity and/or allergy to one or more components of the printed cast, and patients who were not available for follow-up. Patient assessment at presentation to the ED consisted of history taking, physical examination, and confirmation of the suspected injury on radiographic images. Standard treatment consisted of immobilization with either a short arm or thumb spica plaster of Paris cast, depending upon the fracture diagnosis. After providing their informed consent, patients suitable for the current study were invited to attend the orthopedic clinic (inside the hospital) one week after the ED visit to convert the standard cast into a 3D-printed cast. The purpose of the delay of one week was to allow the swelling to subside for optimal adjustment of the printed cast.

### 3D printing workflow

We implemented the workflow as described by Keller et al. [15]. The scanning and modeling were performed by the primary investigator, who is an orthopedic surgeon, and the printing and post-processing steps were conducted by a hospital staff medical engineer. The following is a brief description of the workflow.

#### Scanning

One week after the injury, the affected limb was scanned by a tablet (Apple iPad1 6th generation Apple Inc.TM, Cupertino, California, USA) with an accuracy of approximately 1 mm and an optical structure sensor (Mark I Structure Sensor-1, Occipital Inc.TM, Boulder, Colorado, USA) (Appendix A). The optimal design of the cast (i.e., with or without thumb immobilization) was determined prior to the scanning process of the extremity, which took about 30 s. The wrist position, including flexion and extension and ulnar and radial deviation, could be adjusted following the scan.

#### 3D modeling

In the past, this step had been outsourced to professional designers due to its complexity. It has since become semi-automated using the purpose-built application, Spentys^©^ Point-of-Care Solution^®^ (Spentys SA/NV^TM^, Brussels, Belgium). The main part of the model is automatic, with slight modifications and additions that can be made manually, such as determining the thickness of the cast, adding belt hooks, and adjusting the model to the size of the limb and thumb. Bony prominences (i.e., distal ulna) are addressed as well. The completed model is saved as a stereolithography file, and the entire printing process can be carried out at the hospital.

#### 3D printing

**Figure 1:**
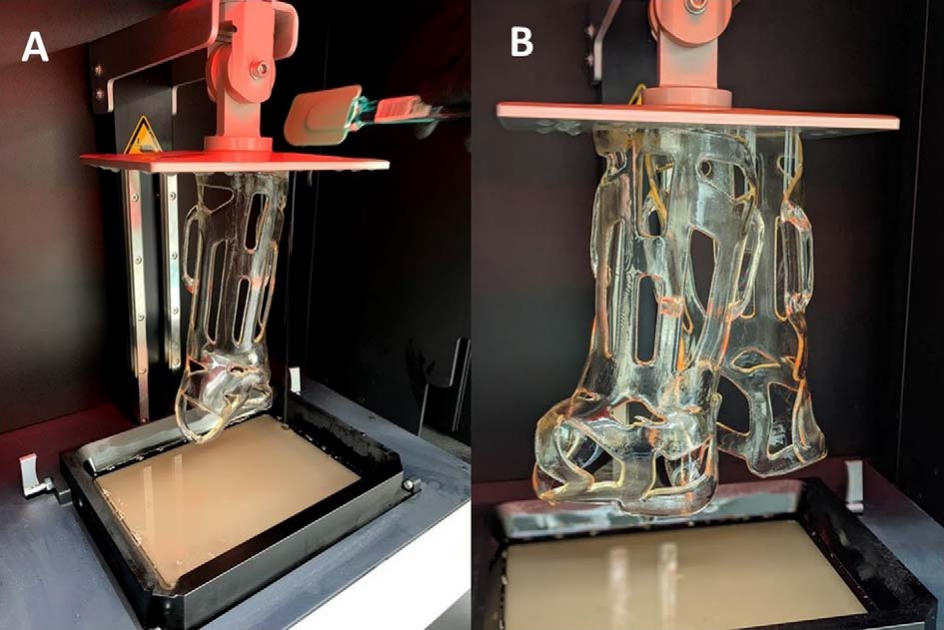
DLP printing: a vertically moving building platform in a tank filled with photosensitive resin, polymerized layer-by-layer by exposure to light (A). Simultaneous printing of two casts (B). With ideal configuration, it is possible to print up to four casts at once without extending the printing time. DLP: digital light processing.

Once the stereolithography file is available online, it can be uploaded with printing instructions onto a 3D printer’s controlling/slicing software (atum3DTM, Gouda, The Netherlands) (Appendix B). Digital light processing (DLP) technology reduced printing and post-processing time [16]. DLP is based upon the polymerization of liquid resin, which becomes solid when exposed to a light source (“cured”) and is formed layer-by-layer (“slicing”) [17]. Handling the unpolymerized resin may cause skin or eye irritation and therefore mandates the use of protective nitrile gloves and protective glasses. The average printing time of a forearm cast was 90 min. According to the manufacturer’s settings, with optimal composition, it is possible to print up to four casts simultaneously without extending the printing time (we printed up to two casts at the same time in this study (Figure 1).

#### Post-processing and fitting

**Figure 2:**
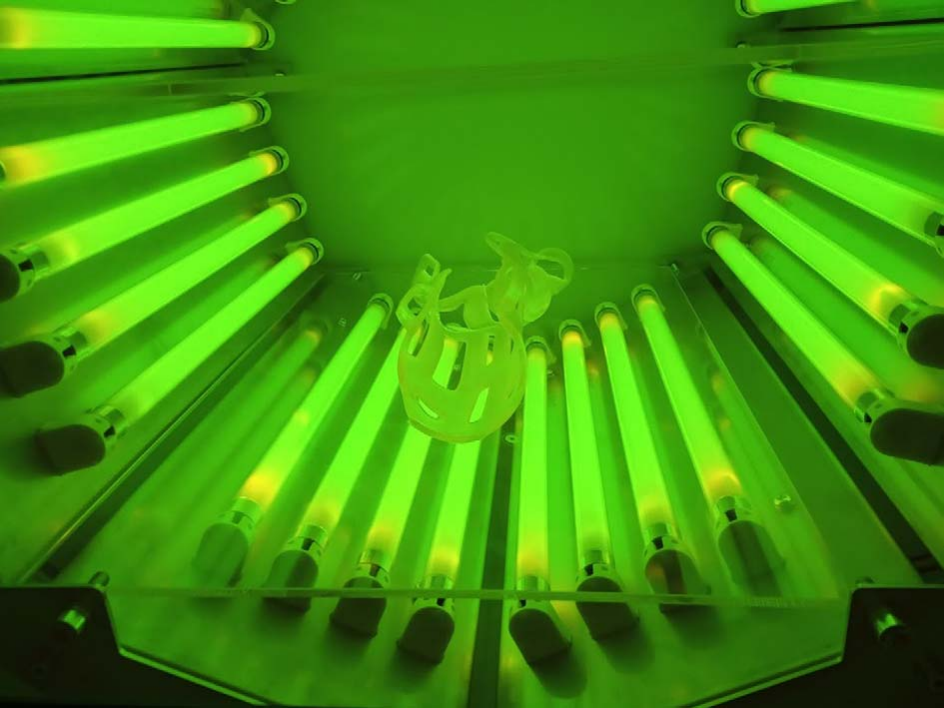
Post-processing of the 3D-printed cast. The cast is placed in a vacuum chamber and exposed to ultraviolet light to verify that all the resin has been cured.

**Figure 3:**
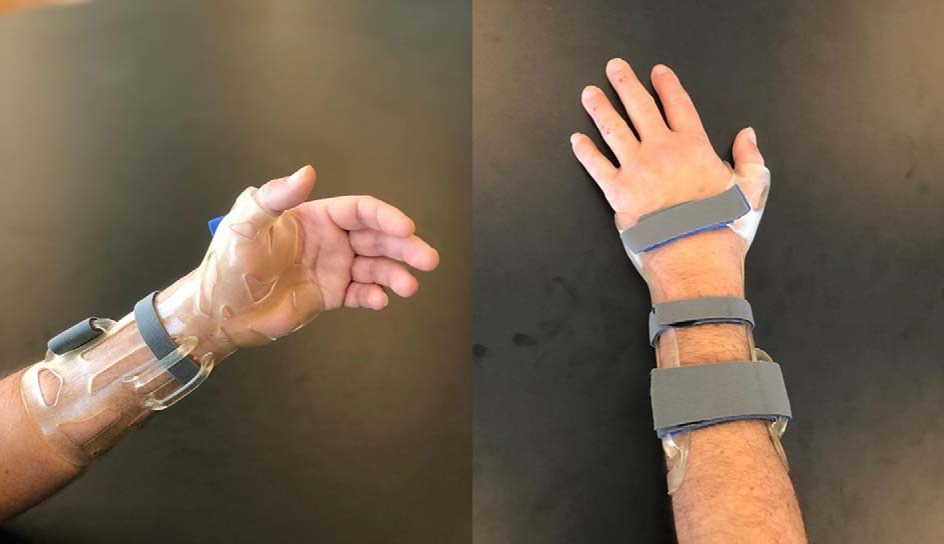
3D-printed thumb spica cast for the treatment of non-displaced scaphoid fracture.

Post-processing is crucial for removing any residual unpolymerized resin before the cast can be fitted to the patient [18]. Cleaning involves spraying concentrated ethanol and placing the printed cast first in an ultrasonic cleaning station and then in a vacuum chamber (Figure 2) exposed to ultraviolet light to verify that all the resin has been cured. (Appendix C). Once the resin is cured, the cast can be put directly onto the skin, with or without a stockinet underneath (according to patient preference). Velcro^©^ fasteners (Velcrotex SATM, Assens, Switzerland) (Figure 3) are used to secure the cast.

### Data collection and clinical outcome measures

Three follow-up examinations and investigations were performed around the second, sixth, and twelve weeks after the application of the 3D-printed cast.

The primary objective was to examine the clinical feasibility of in-hospital production of patient-specific 3D-printed hand casts in terms of complexity and length of the overall procedure. The secondary outcomes were patient-reported outcomes as well as radiological results of the 3D-printed casts in the nonsurgical treatment of wrist and hand fractures.

**Figure 4:**
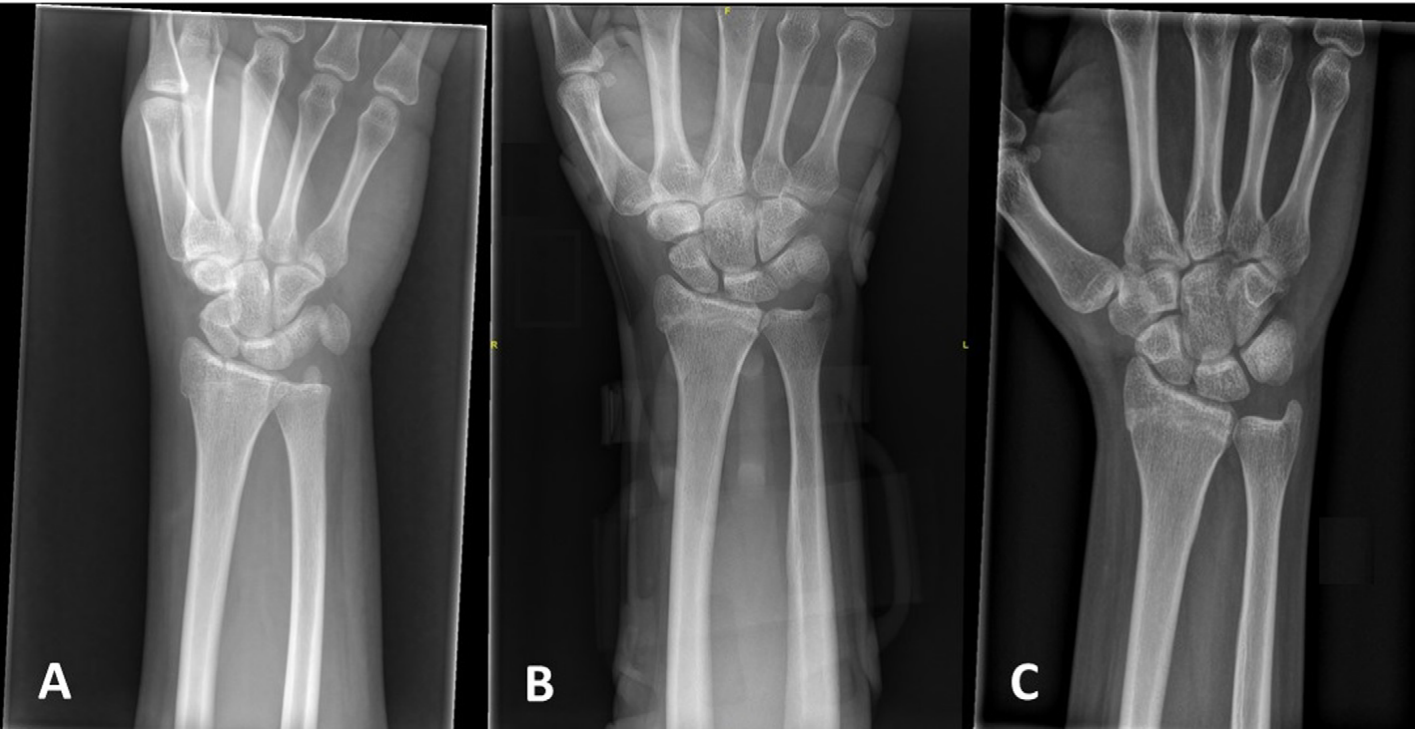
Radiographs demonstrating a radial styloid fracture of the right wrist at presentation to the emergency department. (A). Radiograph at the 6-week follow-up with the 3D-printed cast. Note that the fracture is not concealed, thus allowing for ideal follow-up. (B) Radiograph at 3 months showing union of the fracture with no loss of reduction. (C)

The patient-reported outcome score was assessed by means of the Disabilities of the Arm, Shoulder, and Hand (DASH) score [19]. The pain was assessed with a visual analog scale (VAS) score. Assessment of the clinical effectiveness of the cast was evaluated according to four parameters: stability of immobilization, blood circulation, wear pressure-related pain, and pressure sores [14]. Radiographs of the affected hand were taken to assess secondary displacement of the fracture and signs of the union at 2, 6, and 12 weeks after cast application (Figure 4). Overall, patient comfort and satisfaction were evaluated using a survey consisting of questions associated with treatment-related issues and patient satisfaction assessments [20]. Complications, including failure of the printed cast (i.e., breakage), skin irritation or laceration, displacement, and non-union of the fracture, were recorded.

Twenty suitable patients (16 males) with a mean age of 37 ± 13.1 years (range 20–70) were recruited for this study. There were 13 cases of distal radius fracture, four scaphoid fractures, two cases of hamate fracture, and one case of the base of first metacarpal fracture. The patient demographics are presented in Table 1.

**Table 1:** Patient characteristics.

Characteristic (%)	
Age, mean, years [SD]	37.0 [13.1]
Sex, male	16 (80)
Dominant hand, right	19 (95)
Dominant hand injured	8 (40)
Fractured bone	
Distal radius	13 (65)
Scaphoid	4 (20)
Carpals and MC	3 (15)

SD, standard deviation; MC, metacarpals.

### Statistical analysis

Overall patient characteristics were summarized by mean (±standard deviation) for continuous variables tested for normality or frequency (%) for categorical variables.

## Results

We found it to be simple and effective regarding the clinical feasibility of in-hospital production of patient-specific 3D-printed hand casts. The mean time for the entire printing workflow was 161 ± 8 min (range 146–182). As recommended [15], we conducted preliminary tests before carrying out the process on real patients. Those preliminary steps emerged as crucial for identifying technical problems, refining the process, and improving the efficiency of performance. For example, we found that a cast thickness of less than 3 mm resulted in weaker and excessively flexible casts and therefore set a minimum 3D-printed cast thickness of 3 mm for each patient.

However, there were three cases of cast breakage during the study. It occurred two weeks after application in one patient due to overuse, which included extreme sports activities despite explicit instructions to avoid all strenuous activities during the follow-up period. The breakage occurred in the transition area between the wrist and palm six weeks after applying the 3D-printed cast in the other two cases for no apparent reason. The broken printed casts were re-printed to test their properties and identify any design failure features that could have led to their breakage. No technical problem was noted, and breakage of the re-printed casts occurred when applying direct and extreme pressure. DLP-printed casts are not intended for use in the extreme conditions to which they were submitted by the first patient, just as an ordinary plaster of Paris cast is not made to withstand such activities. In general, the recommendation is to avoid exertion and lifting heavy weights with the casted hand. As for the other two patients, we believe that a 6-week lifespan can be expected on average from the current material (ST45) used with DLP printers. While extensive sun exposure can affect the 3D-printed cast life, an additional 5 min of UV curing may prolong its life span.

As to patient-reported outcome and radiological results, we found that at the 3-month post-injury follow-up, all patients demonstrated good to excellent clinical improvement and fracture union. There were no incidents of pressure sores or loss of reduction among them. The patient comfort and satisfaction rates are presented in Table 2. The mean VAS was 0.9 ± 1.1 and 0.6 ± 1, and the mean DASH score was 18.7 ± 9.5 and 7.6 ± 7.6 at 2 and 6 weeks after application of the 3D-printed cast, respectively.

**Table 2:** Patient satisfaction questionnaire responses rate.

		Totally disagree	Disagree	Agree	Totally Agree
Comfort	You found the splint comfortable			3 (15)	17 (85)
Compliance	You wore the splint the majority of the time / as directed by your doctor			1 (5)	19 (95)
Odor	The splint caused an unpleasant odor	14 (70)	1 (5)	5 (25)	
Itching	You felt the need to scratch	14 (70)	3 (15)	3 (15)	
Itching	You could easily scratch				20 (100)
Ease of use	The splint is easy to put on			2 (10)	18 (90)
Ease of use	The splint is easy to remove	1 (5)		2 (10)	17 (85)
Ease of use	It was easy to put on clothes with the splint			8 (40)	12 (60)
Activities	The splint is annoying during activities	10 (50)	2 (10)	8 (40)	
Weight	The splint is heavy	20 (100)			
Hindrance	The splint is annoying	13 (65)	3 (15)	3 (15)	1 (5)
Adjustment	You quickly adapted to the splint		1 (5)		19 (95)
Durability	The splint breaks quickly	17 (85)	2 (10)	1 (5)	
Cleanliness	The splint quickly becomes dirty	19 (95)		1 (5)	
Water resistance	After contact with water, the limb dries quickly again		3 (15)	2 (10)	15 (75)
Warmth	The limb got warm	18 (90)		1 (5)	1 (5)
Sweat	The limb could sweat		1 (5)	1 (5)	18 (90)
Conformity	The edges of the splint are sharp and harmful	14 (70)	2 (10)	4 (20)	
Conformity	The splint is well shaped to your limb			5 (25)	15 (75)
Satisfaction	You are generally satisfied with the splint			1 (5)	19 (95)
Recommendation	You would recommend the splint to acquaintances			1 (5)	19 (95)

## Discussion

Three-dimensional (3D) printing technology is becoming an increasingly relevant and rapidly advancing tool in medical fields by offering straightforward and cost-effective treatment solutions for clinics and hospitals [1, 10, 21]. Unlike standard casts (i.e., plaster of Paris), a 3D-printed orthopedic cast is tailored to fit perfectly to the patient’s anatomy and pathology, thereby improving patient comfort and satisfaction [11, 12].

In this clinical study, we executed the workflow of in-hospital production of patient-specific 3D-printed devices and applied them to patients with acute wrist or hand fractures. We found it efficient, with excellent clinical and radiographic results and high patient satisfaction and comfort rates.

The mean time of the process from removing the original ED-placed cast to adjusting the 3D-printed cast was 161 ± 8 minutes (range 146–182). Given the average net time of printing (90 min) and an additional 30–40 min of mandatory post-processing curing, we found the in-hospital workflow feasible and relatively fast. In some cases, we printed two casts simultaneously without extending the printing time. With an ideal configuration, it is possible to print as many as up to four casts at once, making the process even more effective [15].

All study participants completed the entire therapeutic course without any adverse events, including loss of reduction, pressure sores, or skin irritation. Patient comfort and satisfaction rates were high, attributed to the 3D-printed cast’s lightweight, breathable, and washable design. Moreover, the 3D-cast features allow daily activities to be performed more easily, as reflected by the excellent DASH scores.

This study has several limitations. It included a relatively small number of patients and a short follow-up period. There were no control groups, thus precluding direct comparisons, although the focus of this study was to investigate the feasibility of the in-hospital workflow and reveal pitfalls in the process. Moreover, there was a selection bias due to the lack of randomization in selecting the patients. However, the heterogeneity among the patients and overall positive patient satisfaction and comfort with the 3D-printed cast reinforces the likelihood that the cast is suitable for most, if not all patients. We included patients with non-displaced fractures, and the application of the 3D casts for displaced fractures has yet to be established. Finally, a medical engineer on our medical center’s staff handled the 3D-designing process: it is not reasonable for a hand surgeon or a hand therapist to carry out this step for every device in a mass-production setting [10].

There are few studies on the use and results of 3D-printing casts since the printing procedure is complex and has been considered impractical in the hospital setting. The only published clinical study was by Chen et al., who reported on 10 patients [14]. Those authors used the mirror technique, which consisted of scanning the counterpart of an injured limb. In the current study, we chose to scan the injured limb one week following the patient’s admission to the ED in order to allow the swelling to subside. In our opinion, that approach offers a more accurate and optimal adjustment of the cast to the injured limb.

## Conclusions

In-hospital workflow for patient-specific 3D-printed casting for the treatment of non-displaced wrist and hand fractures was found to be feasible and efficient, with excellent clinical and radiographic results as well as high patient satisfaction and comfort rates. Additional studies are required to validate our findings, particularly with larger sample sizes and various fracture characteristics.

## Appendix A

### Tablet

Product: iPad 6th Generation (Apple Inc., Cupertino, California, United States).

Operating system: iOS 12, Size: 240 × 169.5 ×7.5 mm, Weight: 469 g, Camera: 8-Megapixel camera, Video recording: 1080p HD video recording.

#### Structure sensor

Product: Mark I Structure Sensor (Occipital Inc., Boulder, Colorado, United States), Max. accuracy: 0.5 mm, Working range: 0.4–3.5 m, Size: 119 × 28 × 29 mm, Weight: 0.1 kg, OS compatibility: iOS.

## Appendix B

### 3D printer

Machine type: atum3D DLP Station 5–405 (atum3D, Gouda, The Netherlands).

Technology: Digital Light Processing (DLP), Max. Build volume: 192 × 108 × 250 mm (W × D × H), Max Print speed: 90 mm/hour, Weight: 37 kg, Print resolution (x, y): 50- or 100-mm. Print resolution Z-axis (layer thickness) 6–500 mm, Projector: Full HD 1920 1080 px, Light source: LED, Wavelength: 405 nm, Ambient operation temperature: 158-328, AC input: 100 × 240 V.

*Resin tray*: exchangeable resin tray with ultra-high chemical resistance which allows third-party and experimental resins. Resin (uncured), Product name: ST45.

Product description: Photopolymer Resin for 3D-printing (SLA, DLP & LCD).

Substances: methacrylic oligomer (65–85%), glycol methacrylate late (10-30%), benzoxazole (<0.05%), phosphine oxide (3.0%) Appearance: viscous liquid – green/turquoise, Odor: ester-like. Melting point: not applicable.

Boiling point: >200 °C Solubility (Water): not soluble, Solubility: good solubility with most organic solvents with relative density: 1.1–1.2 (water = 1). Hazard statements: - H317: may cause an allergic skin reaction. - H319: causes serious eye irritation. - H413: may cause long-lasting harmful effects on aquatic life.

## Appendix C

### Cleaning station

Capacity: 16 L, Technology: Ultrasonic, Total power: 700 W, Max. temperature: 80 °C Bath size: 300 × 300 × 200 mm AC input: 230 V.

#### Curing station

Technology: UV LED & Temperature, LED wavelength: 405/365 nm, LED output: 36 W AC input: 220–240 V.
